# Membrane Distillation: Module Design and Application Performance

**DOI:** 10.3390/membranes16070240

**Published:** 2026-07-16

**Authors:** Lebea N. Nthunya, Bhekie B. Mamba

**Affiliations:** Institute for Nanotechnology and Water Sustainability, College of Science, Engineering and Technology, University of South Africa, Florida Campus, Johannesburg 1709, South Africa; mambabb@unisa.ac.za

**Keywords:** desalination, fouling and scaling, heat and mass transfer, membrane distillation, module design and configuration, resource recovery

## Abstract

Membrane distillation (MD) has emerged as a thermal process used in desalination, wastewater treatment, and resource recovery. It has demonstrated high salt rejection, but process scale-up is affected by unstable process performance caused by membrane fouling and scaling, limitations of heat and mass transfer, and module design challenges. The research outputs presented here assess membrane module design and configurations, hydrodynamic optimization, understanding of membrane fouling, and its control in long-term and intermittent process operation. Furthermore, the integration of crystallization, resource recovery from wastewater, and techno-economic feasibility are also elucidated. The collective findings showed that optimization of the modules, process operating conditions, and the integration of crystallization could improve MD performance stability and resource recovery. Beyond crystallization, MD has demonstrated the ability to recover nutrients from anaerobic digestate, suggesting process expansion directions. These findings provide insights into the key requirements for MD implementation at an industrial scale.

## 1. Introduction

The global scarcity of freshwater is increasing rapidly, threatening the availability of water in the future [1]. Among other factors, water scarcity is exacerbated by human activity, particularly increased generation of industrial wastewater, which is discharged into water bodies such as rivers, dams, and oceans without treatment [2]. If not managed, this lack of freshwater could cause diseases, forced migration, and regional conflicts. This requires integrated solutions for sustainable resource management, driving the development of advanced membrane separation processes [3]. Among other methods, desalination has been extensively explored to increase the supply of water [4,5,6].

One of the emerging technologies used to desalt water is membrane distillation (MD) [7,8]. This has gained research interest due to various advantages such as the utilization of low-grade or waste heat, renewable energy, tolerance to high salinity, and complete rejection of non-volatile compounds [9]. Recent developments have focused on advances in membrane materials, module design, and process optimization [8,10]. These developments have accelerated the MD process’s transition from laboratory and bench-scale investigations to the pilot scale, highlighting the potential implementation direction towards practical industrial applications.

Although recent advancements demonstrate promising solutions, the process remains affected by various scientific and engineering challenges [11,12]. Key performance challenges which affect long-term stability are temperature and concentration polarization (TP and CP), membrane wetting, which reduces separation efficiency, fouling and scaling, module hydrodynamics, high energy consumption, and low energy efficiency [8,13]. These challenges threaten economic feasibility. To compensate for the operational cost of MD, research has expanded towards mineral crystallization for nutrient and resource recovery, promoting zero liquid discharge [14,15].

Although the contributions show technical advances in MD, their industrial implementation remains at an early stage compared with matured desalination technologies such as reverse osmosis (RO). However, considerable achievement has been demonstrated through the benchtop and pilot-scale treatment of seawater, brine, and high-salinity wastewater [16]. The feasibility of pilot-scale and commercial modules has been demonstrated through the incorporation of renewable energy and waste heat to circumvent intense energy demands during water desalination [17]. The current research focuses on niche areas of high salinity, resource recovery, and hybrid systems in pursuit of commercial implementation [18]. Additionally, energy reductions, membrane durability, module design, long-term operational stability and cost of water production form the key strategic areas for the wider industrial deployment of the MD systems [10]. To accelerate MD commercialization, collaboration between academic institutions, industry, and technology developers is essential.

This Special Issue was established to present recent developments in module design, process optimization, fouling control, and resource recovery, paving the way towards industrial implementation of membrane distillation (MD) and membrane distillation crystallization (MDCr). The published studies cover a wide range of experimental investigations, fouling analysis, numerical modelling, resource recovery, and techno-economic assessment, providing detailed current research developments and directions in MD.

## 2. Key Research Findings

### 2.1. Module Design and Process Optimization

Module design remains one of the critical factors governing heat and mass transfer in MD systems. The topic is investigated in two of the studies reported in this Special Issue. Loussif and Orfi (2025) incorporated aluminum metal foam to improve desalination efficiency in Vacuum Membrane Distillation (VMD) [1]. Based on numerical simulation, the insertion of metal foam improved heat transfer and permeate flux and reduced temperature polarization. The highest permeate flux value was reported from the fully inserted foam configuration. However, this increased the pressure drop, suggesting the need to establish a trade-off between heat transfer and hydraulic resistance in high-performance MD systems. Another comprehensive study assessing the influence of hollow-fibre membrane configuration comparison in direct contact membrane distillation (DCMD) was reported by Nthunya and Mamba (2025). The study evaluated parallel and serial connection configurations, assessing the influence of feed temperature, hydrodynamic conditions, cross-flow orientation, and the impact of a scaling agent (MgSO_4_). Both configurations achieved 99% salt rejection, with the permeate quality remaining below 12 µm cm^−1^ throughout the test operation [2]. The parallel configuration exhibited superior long-term stability with reduced concentration and temperature-polarization effects. On the one hand, serial connection demonstrated an earlier onset of supersaturation and crystallization, suggesting suitability in membrane distillation crystallization. The study indicated the importance of module architecture and its influence on transport mechanisms and operation stability. Both Loussif and Orfi (2025) and Nthunya and Mamba (2025) showed that temperature and concentration polarization are governed by module design and configuration. While heat transfer was enhanced by metal foam insertion, the rate of concentration polarization and onset of crystallization are dependent on module connection configuration. Based on these findings, the enhancement of transport efficiency and fouling control depends on process module design.

### 2.2. Membrane Fouling and Scaling

Membrane fouling and scaling remain the main barriers for MD process scaling. To establish mitigation strategies, it is important to understand the mechanisms of fouling and scaling in MD. This phenomenon was covered by two research articles. Cho et al. (2025) investigated membrane fouling during the long-term (120 days) treatment of desalination using hollow-fibre DCMD. The membrane was predominantly fouled by calcium carbonate (CaCO_3_) [3]. Furthermore, fouling was intensified by additional fouling layers caused by the deposition of silica, iron oxides, and organic matter. Fouling was spatial, with intense deposition reported at the feed inlet and inner modules due to concentration and temperature polarization caused by feed supersaturation and scale deposition. The study suggested hydrodynamic optimization to minimize localized fouling in these regions. In another study, Morales et al. (2026) compared the fouling evolution in a continuous vs. intermittent operation during solar-energy-driven desalination via air-gap MD. The highest degree of membrane scaling was reported during intermittent operation caused by CaCO_3_ deposition, and was intensified by nucleation and crystallization due to recurring shutdowns and start-ups [4]. Larger scaling volumes with high cover ratios were reported after membrane cleaning and preservation with deionized water. These operational challenges affect process performance such as permeate quality, stable permeate flux, and mineral resource recovery.

### 2.3. MD Applications for Resource Recovery

MD is experimentally evaluated in resource recovery, which is a logical expansion beyond desalination. This transition was evaluated by Rivera et al. (2025) during the recovery of ammonia (NH_3_) from anaerobic digestate. The study assessed NH_3_ recovery through DCMD, where operational parameters such as pH and thermal gradients were reported to affect nutrient recovery. The resource recovery was predominantly controlled by pH adjustment, where pH 12 and isothermal conditions achieved complete NH_3_ recovery with a molar flux of 1.84 mol TANm^−2^ h^−1^, compared with 36% NH_3_ recovery and a molar flux of 0.63 mol TANm^−2^ h^−1^ at pH 8.2 [5]. These findings demonstrate the dual capability of MD in the valorization of waste-derived streams, ensuring the production of high-purity water and ammonia, used as a fertilizer precursor. Fernandez et al. (2025) integrated DCMD with crystallization for the valorization of oil-produced water. Direct-contact membrane distillation crystallization (DCMDCr) achieved 98% water recovery, ensuring the crystallization of sodium chloride (NaCl) and CaCO_3_ [6]. The composition of recovered salts was 91% NaCl and <5% CaCO_3_. The study further reported techno-economic assessment (TEA) with a DCMDCr of 500,000 gallons day^−1^, demonstrating the enhancement of economic viability through the recovery of by-products and use of waste heat. These studies represent the emerging expansion of MD for applications in water reuse, nutrient and mineral recovery, supporting zero liquid discharge (ZLD), and circular economy frameworks.

## 3. Emerging Research Themes

These reported studies present several emerging themes in MD systems, including the management of hydrodynamic conditions to enhance MD process performance. Key strategies employed to maintain process stability include module connection configuration, controlled flow distribution, and the optimization of heat and mass transfer. The studies further identified membrane fouling and scaling as key hindrances in advancing MD to industrial deployment. Based on reported findings from long-term and intermittent operations, scale formation was induced by the supersaturation and crystallization of scales, intensified at high operating feed temperatures. On the other hand, mineral crystallization plays a critical role in resource recovery, offsetting the challenges of high operational costs in MD systems. The studies demonstrated NH_3_, NaCl and CaCO_3_ recovery from waste streams coupled with production of high-purity water. Prior to MD and MDCr scale-up, techno-economic considerations require further investigation. This is key in ensuring the process transition from laboratory scale to industrial deployment. Furthermore, studies recommend process validation, performance optimization, and the assessment of durability, energy consumption, and detailed economic feasibility to ensure sustainable industrial deployment.

## 4. Future Perspectives

Future research should focus on incremental improvements to advanced module design, MD integration with crystallization, and long-term pilot-scale assessment. Key considerations in module design should ensure process resistance to membrane fouling, temperature, and concentration-polarization effects. In addition, research should focus on intelligent process design and digitization. In addition to module design, research should incorporate the synthesis of three-dimensional (3D) membrane architectures to minimize fouling due to improved flow turbulence. Furthermore, processes evaluated through intermittent operations driven by solar energy should be intensified to establish dynamic fouling control. Similarly, fouling control requires monitoring process and early detection to facilitate control measures. This could be enhanced by integrating artificial intelligence (AI), machine learning (ML), and digital twins. The high cost of MD and MDCr should be countered by the resource recovery of high-value minerals, ensuring sustainable wastewater management. Likewise, further research should intensify the utilization of renewable energy sources such as solar energy, industrial waste heat and low-grade heat to accelerate the global transition towards green energy. For instance, intermittent operations driven by solar energy require control strategies capable of minimizing fouling to ensure stable flux and rejection performance. This could be achieved through autonomous process control which responds to fluctuating thermal gradients in renewable heat. Future studies should also consider integrating other membrane processes such as ultrafiltration (UF), nanofiltration (NF), reverse osmosis (RO) and electrodialysis (ED) to reduce fouling, condition feed water for selective crystallization, and improve process economics. To validate laboratory-scale findings, pilot-scale demonstrations under realistic conditions remain essential. This requires continuous operation for at least 3 to 12 months to determine membrane durability, fouling behaviour, cleaning requirements and membrane ageing. Further research should establish standardized techno-economic and life-cycle assessments which incorporate the comparison between MD, MDCr, and the widely established reverse osmosis (RO) systems.

## 5. Conclusions

The articles published in this Special Issue demonstrate the advancements made regarding MD in desalination, wastewater treatment, and resource recovery. The versatile application of MD has been indicated through advancements in module design, process optimization, fouling assessment, and mineral recovery, ensuring zero liquid discharge. These reported findings demonstrated progressive developments of MD towards sustainability, which is key in addressing the global issues of water, high operational energy demand, and resource recovery.
